# Status of research on the application of melatonin in insomnia based on bibliometric visualization analysis and development trends

**DOI:** 10.3389/fpsyt.2025.1640198

**Published:** 2025-09-29

**Authors:** Huiying Liu, Jinying Zhao, Zhongke Wang, Lu Chen, Yuhong Xie, Ning Yang, Ying Wang, Peng Zheng, Fuchun Wang

**Affiliations:** ^1^ College of Acupuncture and Tuina, Changchun University of Chinese Medicine, Changchun, China; ^2^ Rehabilitation Medicine Academy, Changchun University of Chinese Medicine, Changchun, China

**Keywords:** insomnia, melatonin, neurological disorders, mechanism, VOSviewer, visual analysis

## Abstract

**Objective:**

Insomnia has long been a public health challenge, severely affecting human quality of life. Circadian rhythms are regulated in part by melatonin, and exogenous melatonin has significant therapeutic promise. This study examines global research trends, collaborative networks, and theme evolution in the field of melatonin and insomnia research from 2015 to 2024 using bibliometric visualization techniques.

**Method:**

Using the terms “melatonin” and “insomnia” (as well as their synonyms in the Medical Subject Headings (MeSH) database), this article looked for pertinent literature in the Web of Science Core Collection and PubMed databases. Articles published between January 1, 2015, and December 31, 2024, were included in the search. VOSviewer (version 1.6.20) was used to visualize countries, institutions, journals, and authors; Citespace (version 6.3 R1) was used to visualize keywords and references.

**Results:**

The two databases yielded 1,818 papers in total, of which 1,084 were included following screening. Even though there are annual variations, the general trend is upward. The institutions and nations with the most publications among them are Harvard Medical School and the United States. The author with the most publications is Bruni O. The most significant co-citation frequency is found in Morin C.M. Among the cited references, Gringas P. (2017) has the most citations. Frontiers in Psychiatry and Sleep Medicine are the journals with the most publications.

**Conclusion:**

Melatonin has been used mainly to treat insomnia in children, the elderly, and people with neurological disorders during the last ten years. Further, a trend for future research in this area is the convergence of insomnia with comorbidities like neurological disorders, cancer, and pain medication.

## 1 Introduction

Insomnia is characterized by difficulty falling asleep, waking up during the night, waking up too early, and fatigue during the day. The World Health Organization (WHO) reported that insomnia affects 27% of people worldwide, with a prevalence rate of 38.2% among Chinese adults (1). Obesity, high blood pressure, and heart disease are all directly linked to insomnia (2). In extreme circumstances, it may cause cognitive impairment and precipitate the development of additional neurological conditions (3). It has a major negative influence on people’s quality of life, forcing families and society to bear heavy financial and psychological costs. There are two categories of current insomnia treatment methods: pharmacological and non-pharmacological. Acupuncture, cognitive-behavioral therapy, and traditional Chinese medicine are examples of non-pharmacological treatments, whereas hypnotic and sedative drugs are the mainstay of pharmaceutical treatments.

The pineal gland is where melatonin is mostly produced (4). The pineal gland is linked to the suprachiasmatic nucleus (SCN) through a polysynaptic sympathetic nerve pathway. Light signals are detected by photoreceptive ganglion cells (ipRGCs) in the retina and sent to the SCN, which subsequently sends them to the paraventricular nucleus (PVN) of the hypothalamus through gamma-aminobutyric acid (GABA) neurons. To reach the preganglionic sympathetic neurons, whose axons ascend to the superior cervical ganglion (SCG) for synapse, the descending PVN fibers pass through the brainstem and spinal cord (T1-T3). Postganglionic fibers then innervate the pineal gland (5, 6). High-affinity G protein-coupled receptors called MT1 and MT2 are involved in a variety of neurotransmitters and neurotransmitter systems that regulate sleep. Melatonin, commonly referred to as the “sleep hormone,” is a key regulator of the sleep/wake cycle. A promising treatment strategy for sleep disorders is the selective modulation of these receptors (7). With longer peaks in winter and shorter peaks in summer, melatonin production at night fluctuates with the seasons (8). Melatonin is primarily responsible for controlling blood pressure, cortisol secretion, body temperature, sleep-wake cycles, and circadian rhythms in humans (9).

Scientific studies on melatonin as a complementary treatment for insomnia have expanded quickly in recent decades, but there hasn’t been a thorough bibliometric analysis to map the global research landscape, pinpoint important contributors, and uncover new trends. The goal of this study is to clarify the evolution of melatonin and insomnia research over the last ten years and offer references for further research by visualizing collaboration, thematic clusters, and temporal changes in the field using VOSviewer and CiteSpace. Vosviewer can use co-citation data to construct visual analyses of countries, institutions, authors, or journals (10). CiteSpace is a software designed for the visual analysis and exploration of scholarly literature across various research fields or disciplines (11, 12).

## 2 Materials and methods

### 2.1 Study design

This study aims to map out the global research landscape on melatonin in the field of insomnia research from 2015 to 2024, following the BIBLIO list of bibliometric studies.

### 2.2 Data sources

Two databases were searched: Web of Science Core Collection (WoSCC) and PubMed.

### 2.3 Search strategy

A comprehensive search strategy was developed for each database. See **Supplementary File 1** for details.

### 2.4 Inclusion criteria

(1) Literature published between January 1, 2015, and December 31, 2024.
(2) English-language literature.
(3) Literature type: original research articles and reviews related to melatonin and insomnia.

### 2.5 Exclusion criteria

Conference proceedings, abstracts, editorial materials, letters, collections, anthologies, news items, book chapters, and retracted publications.

### 2.6 Screening process

**Figure 1:**
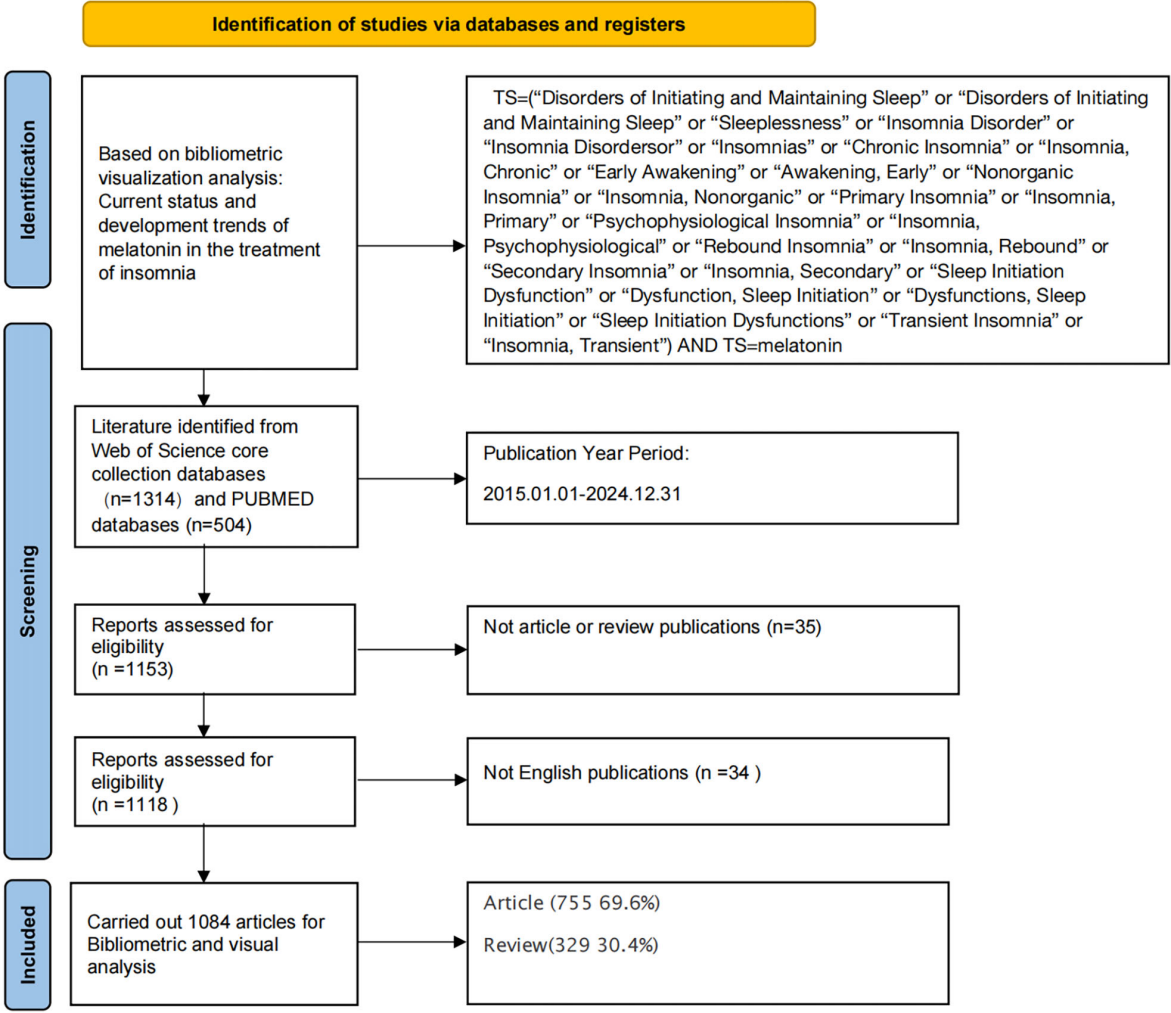
Literature screening flowchart.

Two independent reviewers screened all retrieved records based on titles, abstracts, and full texts. Disagreements were resolved through consensus or by a third reviewer. A PRISMA-style flowchart (**Figure 1**) summarizes the screening process, including the records retrieved from each database and the stepwise exclusion steps, ultimately determining the final set of included literature.

### 2.7 Data extraction and processing

Data is exported from the database in the “txt” format, with file names that start with “download_*.txt.” Citespace (6.3R1) and Vosviewer (1.6.20) tools are then used to analyze the files and search for keywords, references, publications, nations, institutions, journals, co-cited journals, authors, and co-cited authors.

CiteSpace’s parameters were set as follows: (1) 2015–2024-time frame; (2) node type selection at a time; (3) g-index selection of k-value set to 25; and (4) Top N option set to 50. Nodes in a CiteSpace network knowledge graph stand for the informational unit of the object under study, and node sizes indicate how frequently an event occurs. The time and quantity of the examined items are indicated by the color and breadth of the “year rings” inside the nodes. On the other hand, a high degree of intermediate centrality throughout the whole network is shown by the purple outside rings. The degree of correlation between the various factors under analysis is shown by the thickness of the connecting lines.

### 2.8 Metrics for analyzing literature

(1) Analysis of annual publication trends.
(2) Analysis of countries, institutions, journals, co-cited journals, authors, co-cited authors, references, and keyword co-occurrence.
(3) Cluster analysis of research hotspots and future trends.

## 3 Results

### 3.1 Analysis of annual publications

**Figure 2:**
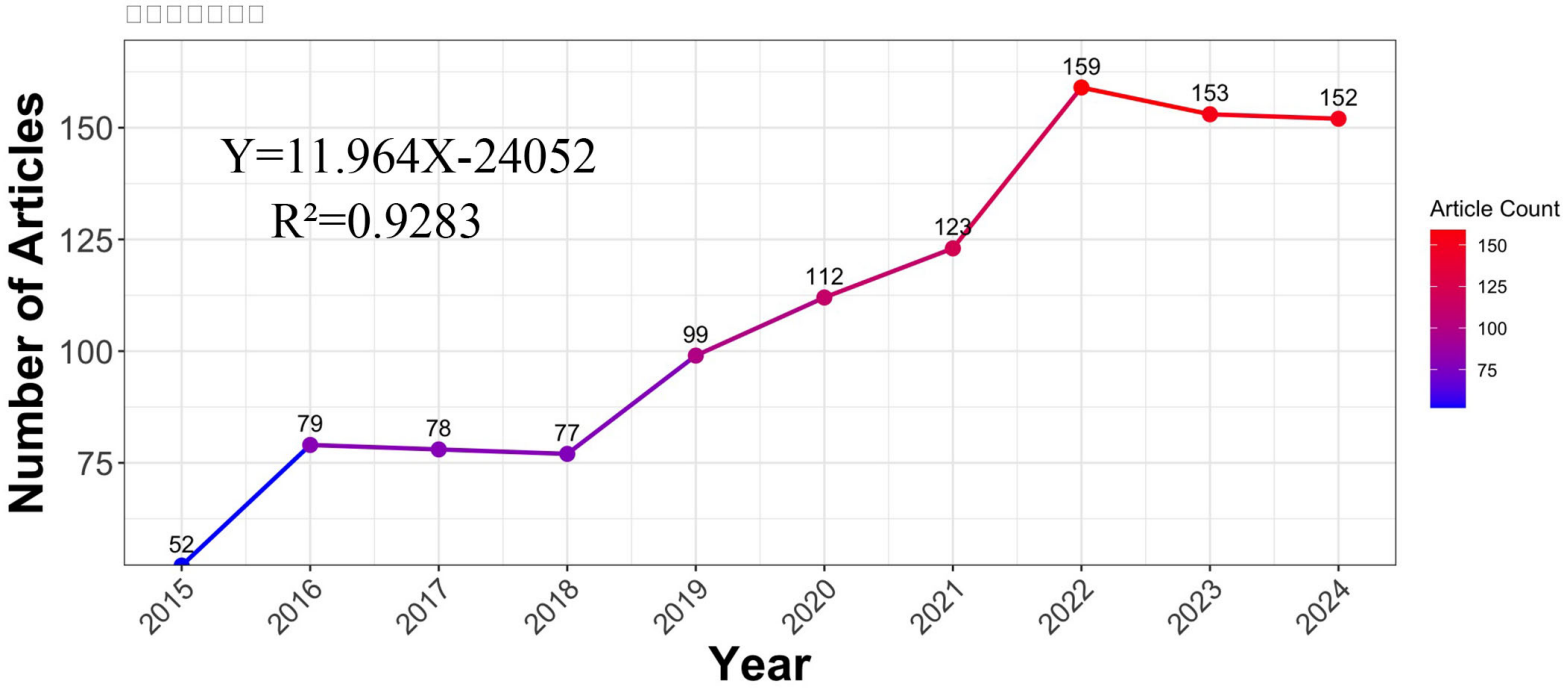
Number of publications on melatonin in insomnia.

Since it represents the general trend of development in this field of study, the number of publications per year is very important. The publication volume of research articles about melatonin and insomnia over the last ten years can be broken down into three phases, as seen in **Figure 2**. Although there were comparatively few publications during the first phase, which lasted from 2015 to 2018, there was an overall upward trend. The number of publications steadily rose from 99 to 159 during the second phase, which spanned 2019 to 2021. The number of publications peaked during the third phase, which ran from 2022 to 2024. In 2022, the number of articles about melatonin and insomnia peaked at 159, making it the year with the most publications in this area in the previous ten years. A linear trend line was created for annual publications in order to better understand the output trend. This resulted in the equation Y=11.964X-24052, where Y stands for annual publications and X for the year. The coefficient of determination (R²) for the model is 0.9283. Overall, the number of articles about melatonin’s role in insomnia has been rising steadily, which is indicative of the topic’s increasing significance and popularity.

### 3.2 Analysis of country co-occurrence networks

**Figure 3:**
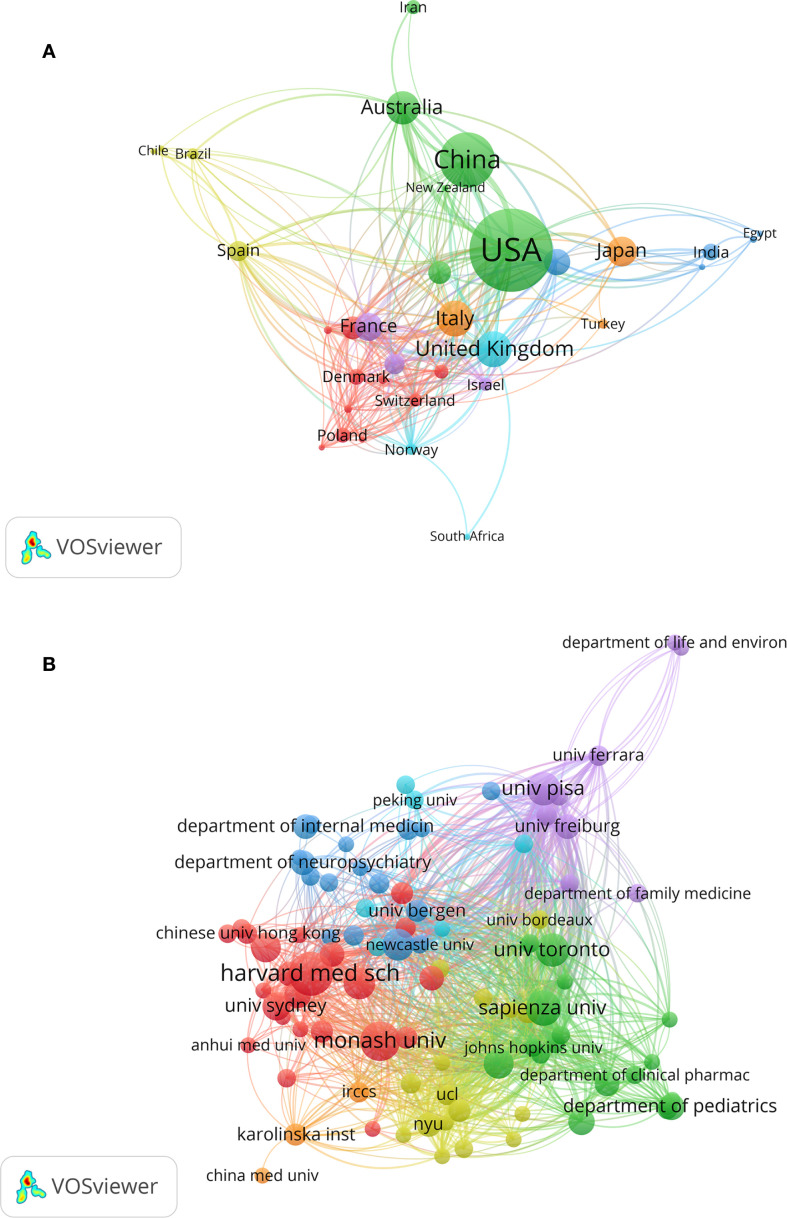
National cooperation network map **(A)** and institutional cooperation network map **(B)**.

Open VOSviewer 1.6.20, Import the search results, and examine the countries’ co-occurrences. Research on this subject was conducted in 428 different countries. A national-level collaboration network with a minimum of five publications is depicted in **Figure 3A**. With a total link strength of 900, the results show a co-occurrence map of 34 countries, demonstrating strong international cooperation. With a total link strength of 216, the United States is at the center of the collaboration network and demonstrates extensive international collaboration. The United Kingdom and the United States have the closest collaboration with other nations. With a total link strength of 153, the UK demonstrates high centrality as well. Comparable node sizes between China and the US suggest similar publication volumes. The network has seven clusters, including the Asia-Pacific cluster, which is represented by China, Australia, and Japan, and the European cluster, which is represented by France, Italy, and Switzerland.

**Table 1** highlights the top 10 countries by number of publications and looks at their publication volume, citation rates, and link strength. In terms of publications, the United States leads with 300, followed by China with 167 and the United Kingdom with 90. The United States received 8,153 citations, followed by the United Kingdom (3,968) and Italy (3,286). Germany had the highest average citation rate (46.02), followed by the UK (44.01) and France (43.69). The United States dominates this field of study, as evidenced by the national analysis, which shows that it leads in both publications and citation rates. China has 167 publications, which puts it in second place, but its citation rate is lower than that of other countries, indicating that output volume and research influence are not always related. In terms of average citation rates, European nations—including the UK, Germany, Italy, and France—perform better than Asian nations.

**Table 1:** Top 10 countries by publication volume.

Rank	Country	Documents	Citations	Total link strength	Average citations
1	USA	300	8153	216	27.18
2	China	167	2091	82	12.52
3	United Kingdom	90	3968	153	44.01
4	Italy	88	3286	122	37.34
5	Australia	79	1496	90	18.94
6	Japan	69	828	30	12
7	France	64	2796	120	43.69
8	Canada	56	2344	87	41.86
9	South Korea	47	486	21	10.34
10	Germany	46	2117	110	46.02

### 3.3 Network analysis of institutional collaboration

A total of 2,465 institutions contributed to the publication of articles on this subject, according to the data that was retrieved and imported into Vosviewer 1.6.20 for institutional analysis. The collaborative network at the institutional level is depicted by **Figure 3B**, which shows 92 institutions. The institutions’ close cooperation is indicated by the total link strength of 3,382. Sapienza University, University of Pisa, University of Southampton, Johns Hopkins University, and University Hospital of Strasbourg are among the top five universities based on link strength analysis, which shows that their links are thicker. Europe has a cluster advantage in melatonin research, as evidenced by the fact that three of these institutions are European. Harvard Medical School (692) is in second place, followed by the University of Surrey (634) in third place, and the University of California, San Francisco (1079) is at the top among citation rates.

Each of the top 10 institutions in terms of publication volume has published more than 11 papers, as shown in **Table 2**. The top three universities are Harvard Medical School (21 publications), Monash University (18 publications), and Sapienza University (15 publications). A total of 143 articles were published by these top 10 institutions, making up 13.2% of all publications. It is noteworthy that Harvard Medical School has a high average citation rate and publication volume.

**Table 2:** Top ten institutions in terms of publication volume.

Rank	Organization	Country	Documents	Citations	Total link strength	Average citations
1	Harvard Medical School	USA	21	692	108	32.95
2	Monash University	Australia	18	469	113	26.05
3	Sapienza University	Italy	15	411	374	27.4
4	University of Pisa	Italy	14	276	279	19.71
5	University of Toronto	Canada	14	458	66	32.71
6	University of Surrey	United Kingdom	13	634	128	24.07
7	University of Copenhagen	Denmark	13	313	114	48.77
8	University of Southampton	United Kingdom	12	477	241	39.75
9	Stanford University	USA	12	629	32	52.42
10	University of Sydney	Australia	11	201	80	18.27

### 3.4 Journal collaboration network analysis

**Figure 4:**
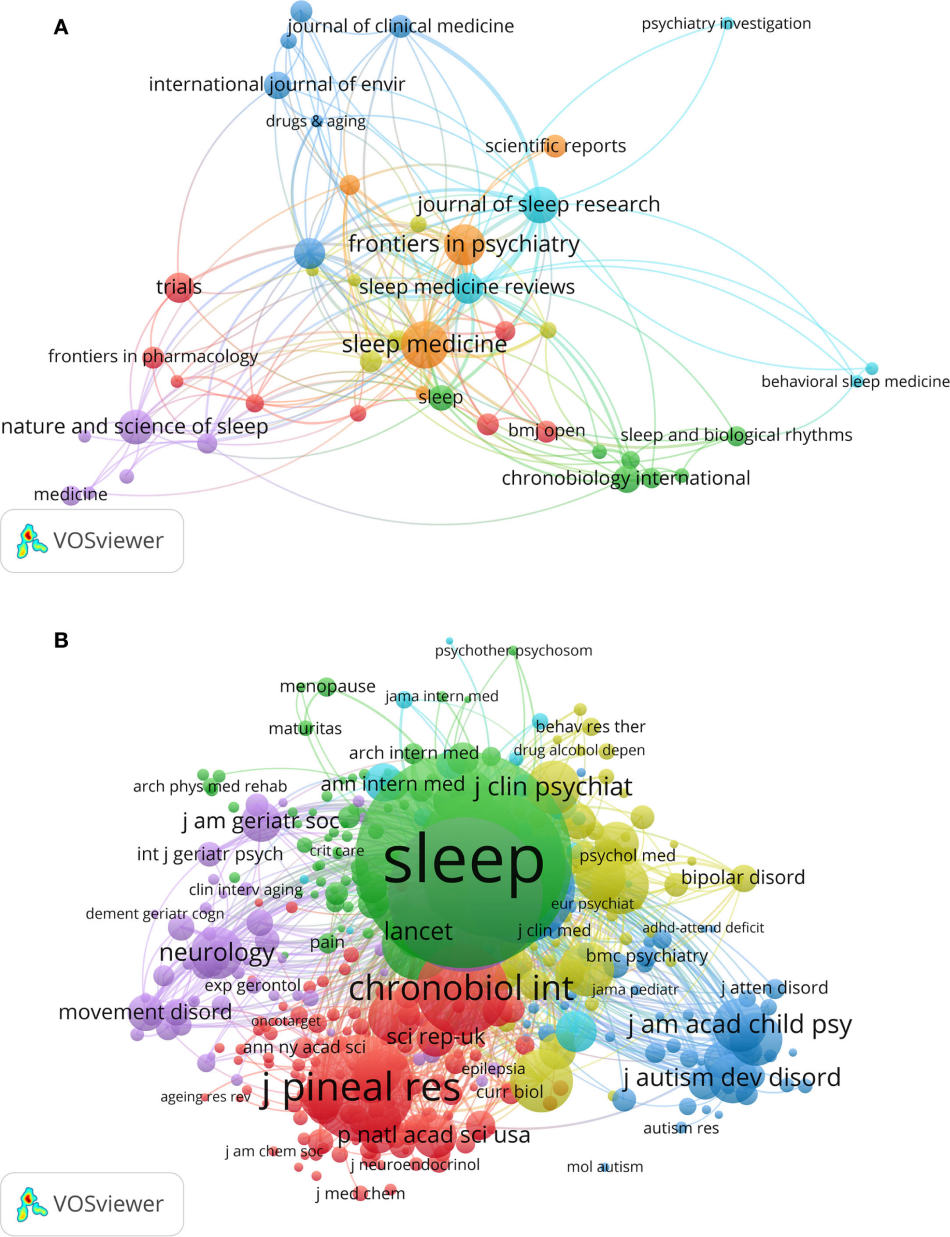
Journal cooperation network map **(A)** and cited journal network map **(B)**.

For journal analysis, the obtained data was imported into Vosviewer 1.6.20, which revealed that 488 journals had published these kinds of articles. **Figure 4A** shows the collaboration networks of 43journals. In total, the top 10 journals published 185 papers, or 17.07% of all publications, as shown in **Table 3**. Out of all of these, SLEEP MEDICINE ranked first and published the most studies (n = 31). Frontiers in Psychiatry came in second with 25 studies, and JOURNAL OF SLEEP RESEARCH came in third with 21 studies. Indicating that the caliber of publications in this field of study is low, two of the top ten journals had Impact Factors (IF) higher than five, while the average for the remaining journals was three. Of these, the journal Sleep Medicine Reviews is at the forefront of its field, as evidenced by its highest IF of 11.2 and Q1 zone ranking in the JCR classification. This indicates that the journal is well-known among researchers and has a high degree of academic standards and influence in its field. With a JCR classification ranking in the Q1 zone and an IF of 5.3, SLEEP comes next.

**Table 3:** Top ten journals in terms of publication volume.

Rank	Source	IF	JCR	Documents	Citations	Total link strength	Average citations
1	SLEEP MEDICINE	3.8	Q1	31	680	65	21.94
2	Frontiers in Psychiatry	3.2	Q2	25	252	33	10.08
3	JOURNAL OF SLEEP RESEARCH	3.4	Q2	21	1572	61	74.86
4	Nature and Science of Sleep	3.0	Q2	20	183	16	9.15
5	Journal of Clinical Sleep Medicine	3.5	Q1	17	1086	44	63.88
6	SLEEP MEDICINE REVIEWS	11.2	Q1	17	1147	68	67.47
7	Trials	2.0	Q3	16	59	8	3.69
8	CHRONOBIOLOGY INTERNATIONAL	2.2	Q3	14	344	10	24.57
9	SLEEP	5.3	Q1	13	311	24	23.92
10	Journal of Clinical Medicine	3.0	Q1	11	63	15	5.73

Examine the co-cited journals after importing the search results into VOSviewer 1.6.20. This suggests that a total of 6,096 journals is writing about this subject. **Figure 4B**: Nodes that are cited sources in the research field literature are represented by the 399 journals that are shown. The number of citations is indicated by the size of the node, the larger the node, the greater the influence and the number of citations. A stronger co-citation relationship is indicated by thicker connecting lines, which show how frequently two journals are cited together in the same article. Six clusters are created from the data; these clusters show the outcomes of the journal citation clustering process. Similarly colored journals tend to represent a discipline or field and have more similar citation patterns. The top ten journals in terms of co-citation frequency are listed in **Table 4**. Among these, Sleep has the highest total citation rate (2,310), followed by Sleep Medicine (1,414) and Sleep Medicine Reviews (1,295). Indicating that the field is still in a steady accumulation phase, the majority of impact factors are concentrated in the 3–5 range. The fact that Sleep Medicine Reviews has a high single-article influence but a small publication volume is reflected in its impact factor of 11.2 and third-place citation rate. Due to their higher publication volumes, Sleep and Sleep Medicine achieve higher total citation counts, despite having impact factors of 5.3 and 3.8, respectively.

**Table 4:** Top ten journals ranked by citation rate.

Rank	Source	IF	JCR	Citations	Total link strength
1	SLEEP	5.3	Q1	2310	152477
2	SLEEP MEDICINE	3.8	Q1	1414	93155
3	SLEEP MEDICINE REVIEWS	11.2	Q1	1295	89022
4	JOURNAL OF SLEEP RESEARCH	3.4	Q2	850	56690
5	JOURNAL OF PINEAL RESEARCH	8.3	Q1	835	50919
6	Journal of Clinical Sleep Medicine	3.5	Q1	782	50390
7	CHRONOBIOLOGY INTERNATIONAL	2.2	Q3	660	38804
8	PLoS One	2.9	Q1	477	30250
9	JOURNALOF BIOLOGICAL RHYTHMS	2.9	Q2	393	23753
10	JOURNAL OF AFFECTIVE DISORDERS	4.9	Q1	344	23018

### 3.5 Author collaboration network analysis

A total of 4,854 authors took part in this study, according to the data imported into VOSviewer 1.6.20 for author analysis. The top ten authors and the authors who receive the most citations are listed in **Table 5** according to the number of articles they have written. Of them, Ferri R and Zisapel N tied for third place with 11 articles each, while Bruni O leads with 17 articles, followed by Palagini L with 15. Citation counts showed that Zisapel N had the most citations (645), followed by Bruni O (551) in second place and Malow B. A (347). in third. Interestingly, Bruni O has a high citation rate and a large number of publications, which suggests that her work is of high quality in this field of study.

**Table 5:** Top ten authors ranked by publication volume and citation rate.

Rank	Author	Documents	Author	Citations
1	Bruni Oliviero	17	Zisapel Nava	645
2	Palagini Laura	15	Bruni Oliviero	551
3	Ferri Raffaele	11	Malow Beth A.	347
4	Zisapel Nava	11	Gringras Paul	336
5	Biggio Giovanni	10	Cortese Samuele	313
6	Cortese Samuele	10	Nir Tali	310
7	Inada Ken	10	Breddy John	310
8	Mishima Kazuo	10	Ferri Raffaele	284
9	Riemann Dieter	10	Skene Debra J.	282
10	Gringras Paul	9	Rajaratnam Shantha M. W.	253

**Figure 5:**
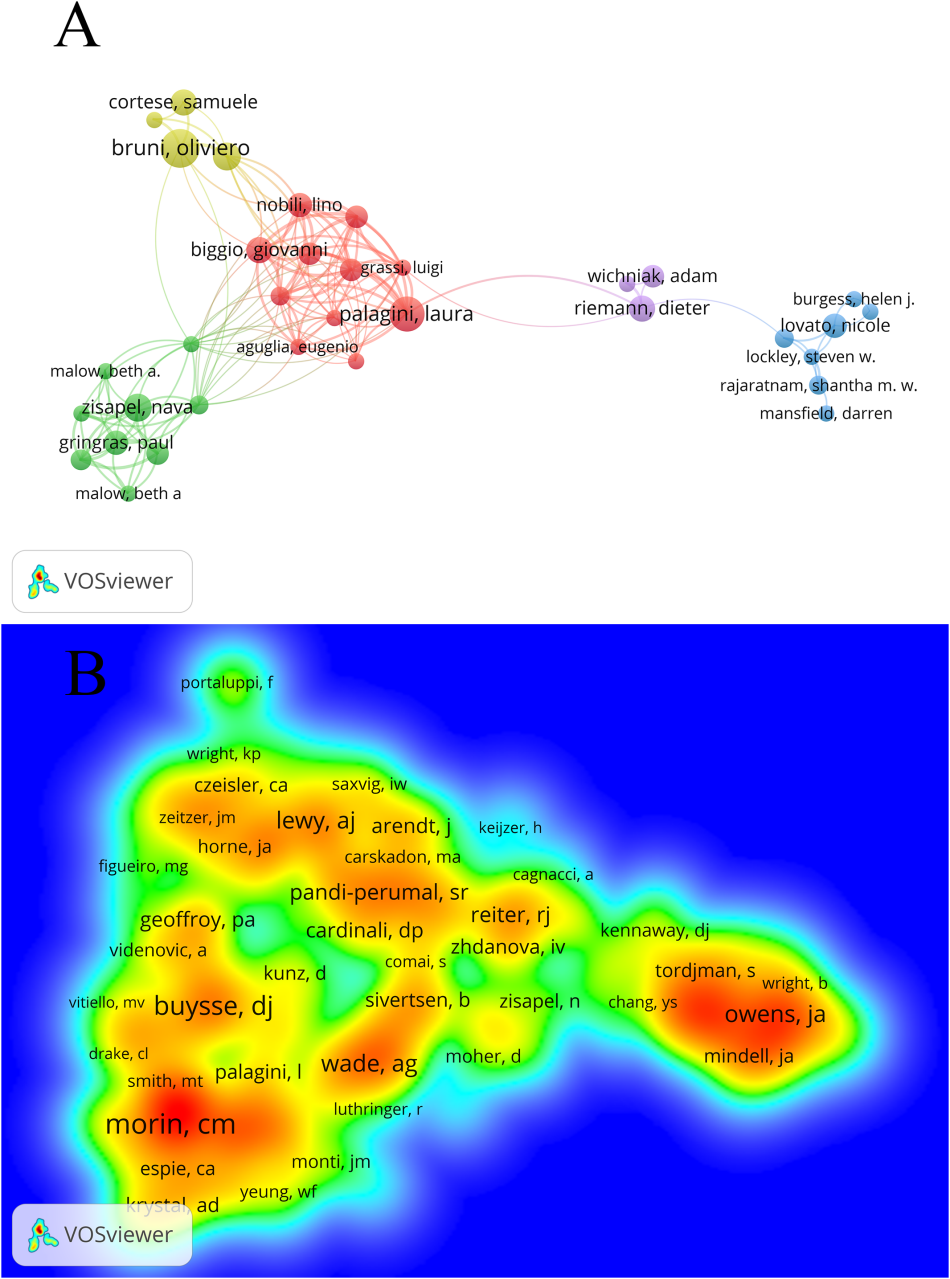
Author cooperation network map **(A)** and co-cited author network map **(B)**.

**Figure 5A** illustrates author collaboration by grouping 34 authors into 5 clusters. Aguglia E, Biggio G, Geoffroy P. A., Girardi P, Grassi L, Manni R, Minervino A, Nobili L, Palagini L, Philip P and Plazzi G are among the authors working together in the same cluster. The grouping of co-cited authors is shown in **Figure 5B**. The brightness of each color corresponds to the frequency of citations; the higher the frequency, the brighter the hue. This allows for the easy identification of the most frequently cited authors among the 21,350 co-cited authors, of which 232 are displayed. The author with the closest ties to other authors is Morin C.M.

### 3.6 Keyword analysis

**Figure 6:**
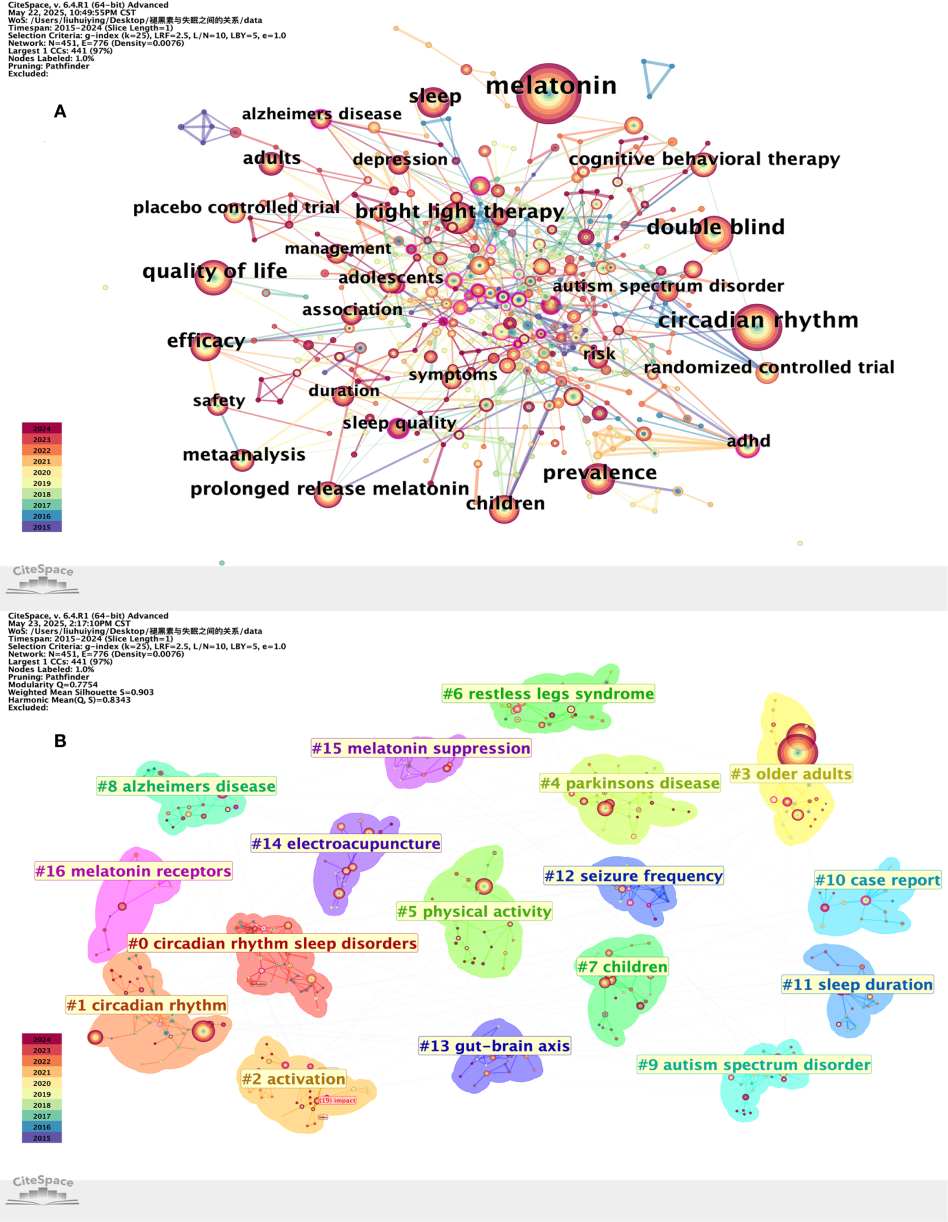
Keyword co-occurrence map **(A)** and keyword clustering map **(B)**.

A keyword visualization analysis was performed using Citespace. This study’s co-occurring keywords visualization analysis diagram has 451 nodes and 776 links, with a network density of 0.076 (**Figure 6A**). Sleeplessness (475), melatonin (279), circadian rhythm (166), double blindness (111), quality of life (100), bright light therapy (90), prevalence (85), sleep (79), efficacy (71), and children (61) are among the top 10 keywords. “Insomnia” was the most frequently occurring of the high-frequency keywords found, with 475 occurrences, indicating its importance as a worldwide health concern in sleep medicine research. The three primary components of the research network, “circadian rhythm” (166 occurrences) and “melatonin” (279 occurrences), came in second and third, respectively, underscoring the crucial role of the melatonin system in insomnia. According to frequency analysis, insomnia is still a global problem and a research hotspot, as **Table 6** illustrates, with “body temperature” having the highest centrality (0.16). An important focus area in the field is represented by a node with a centrality value >0.1, which denotes the node with the greatest attention and influence in the research field. In the future, body temperature rhythms could be a novel approach to treating insomnia because they are a significant biomarker of circadian rhythm function and have special significance in diagnosing insomnia that is disordered by the circadian rhythm. Additional keywords that have centrality values greater than 0.1 are “double-blind” (0.12) and “quality of life” (0.11), which highlight the significance of patient-reported outcomes and methodological rigor in this area.

**Table 6:** Top ten keywords by frequency and centrality ranking.

Rank	Freq	Keywords	Rank	Centrality	Keywords
1	475	insomnia	1	0.16	body temperature
2	279	melatonin	2	0.14	adhd
3	166	circadian rhythm	3	0.14	controlled trial
4	111	double blind	4	0.14	major depressive disorder
5	100	quality of life	5	0.14	phase syndrome
6	90	bright light therapy	6	0.14	adjunctive ramelteon
7	85	prevalence	7	0.13	alzheimers disease
8	79	sleep	8	0.12	sleep quality
9	71	efficacy	9	0.12	chronic insomnia
10	61	children	10	0.12	bipolar disorder

### 3.7 Keyword clustering analysis

The keyword cluster visualization analysis is displayed in **Figure 6B** after the data was imported into Citespace (version 6.3 R1). As demonstrated by the results, the modularity Q-value has a distinct and significant clustering structure, surpassing the 0.3 threshold at 0.697. Furthermore, it was found that the clusters’ average contour value was 0.86, suggesting that this unique clustering technique is dependable and reasonable. A total of seventeen clusters were produced using various clustering labels: #0 circadian rhythnm sleep disorders, #1circadian rhythnm, #2activation, #3older adults, #4parkinsons disease, #5physical activity, #6restless legs syndrome, #7children, #8alzheimers disease, #9autism spectrum disorder, #10case report, #11sleep duration, #12seizure frequency, #13gut-brain axis, #14electroacupuncture, #15melatonin suppression, #16melatonin receptors.

Classify the above keyword cluster tags, where tags such as #0 circadian rhythm sleep disorders, #4 Parkinson’s disease, #6 restless legs syndrome, #8 Alzheimer’s disease, and #9 autism spectrum disorder are associated with insomnia comorbidity. Tags such as #1 circadian rhythnm, #13 gut-brain axis, #15 melatonin suppression, and #16 melatonin receptors are related to the mechanisms of insomnia. #2 activation, #14 electroacupuncture, and #5 physical activity are related to intervention or treatment methods. #3 older adults and #7 children are related to the population with insomnia. #10 case report and #11 sleep duration are outcome measures related to insomnia.

### 3.8 Analysis of cited references

**Figure 7:**
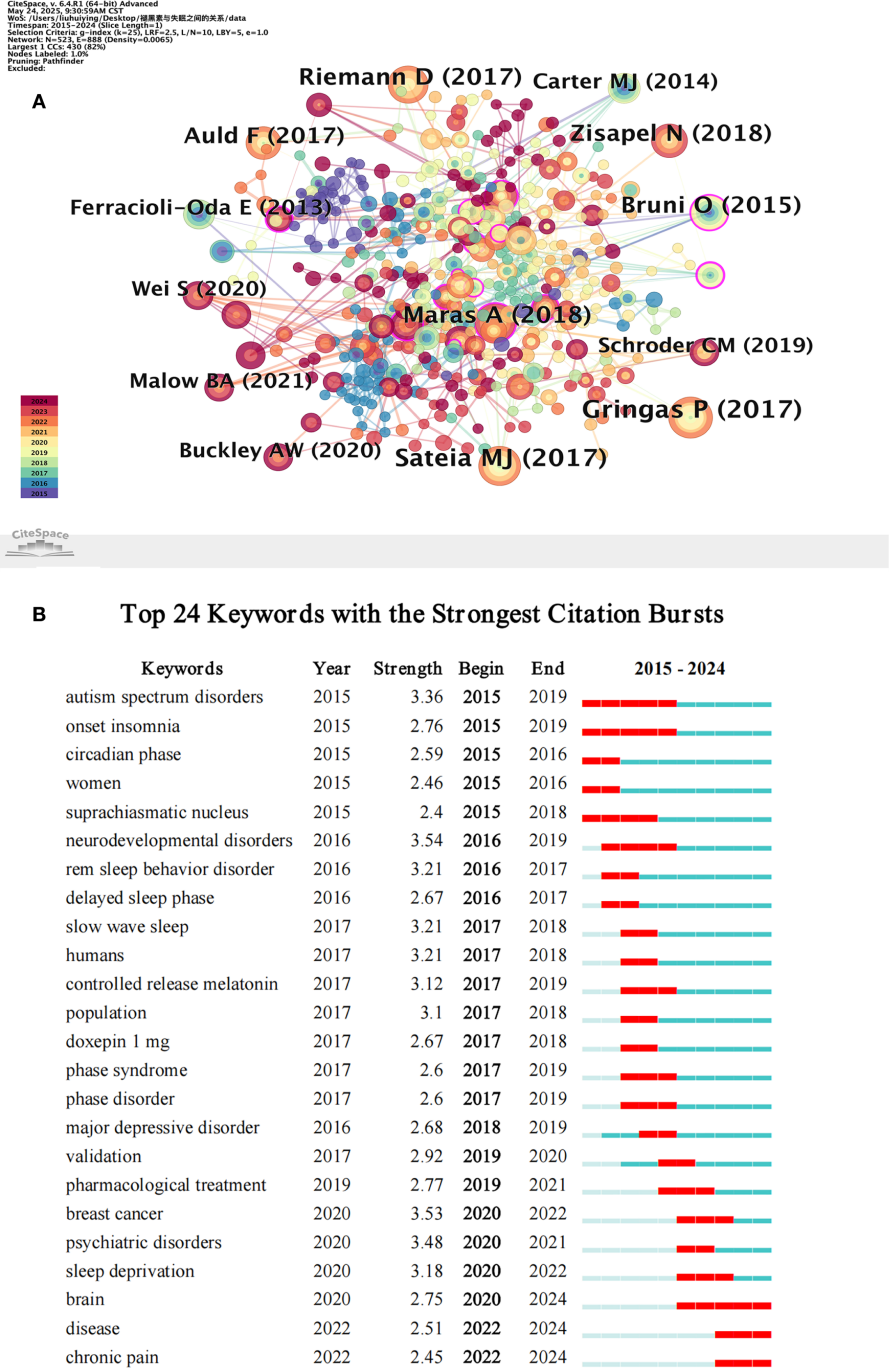
Co-occurrence Map of References **(A)** and the 24 most frequently cited keywords **(B)**.

Select the cited references for visualization analysis after importing the data into CiteSpace (version 6.3 R1). **Figure 7A** illustrates this, with a network density of 0.0108 and 249 nodes and 333 links. Gringas P. (2017), Sateia M.J. (2017), Maras A. (2018), Riemann D. (2017), Zisapel N. (2018), and Bruni O. (2015) are among the top five cited references by citation count, as shown in **Table 7**. Elrod MG, 2015 (0.19), Bruni O, 2018 (0.17), Goldman SE, 2014 (0.21), Chang YS, 2016 (0.29), and Auger RR, 2015 (0.17), are the top five references in terms of centrality, as shown in **Table 8**. Chang YS, 2016 (0.29), which was published in Jama Pediatrics, is noteworthy for having a high article quality, ranking first in centrality, having an IF of 24.7, and being in JCR Zone 1.

**Table 7:** Top five most cited references.

Rank	Frequency	Cited reference	IF	Journal
1	35	Efficacy and Safety of Pediatric Prolonged-Release Melatonin for Insomnia in Children with Autism Spectrum Disorder	9.2	Journal of The American Academy of Child and Adolescent Psychiatry
2	33	Clinical Practice Guideline for the Pharmacologic Treatment of Chronic Insomnia in Adults: An American Academy of Sleep Medicine Clinical Practice Guideline	3.5	Journal of Clinical Sleep Medicine
3	28	European guideline for the diagnosis and treatment of insomnia	3.4	Journal of Sleep Research
4	28	Long-Term Efficacy and Safety of Pediatric Prolonged-Release Melatonin for Insomnia in Children with Autism Spectrum Disorder	1.5	Journal of Child and Adolescent Psychopharmacology
5	26	New perspectives on the role of melatonin in human sleep, circadian rhythms and their regulation	6.8	British Journal of Pharmacology
5	26	Current role of melatonin in pediatric neurology: clinical recommendations	2.3	European Journal of Paediatric Neurology

**Table 8:** Top 5 references by centrality.

Rank	Centrality	Cited reference	IF	Journal
1	0.29	Melatonin Supplementation for Children with Atopic Dermatitis and Sleep Disturbance: A Randomized Clinical Trial	24.7	Jama Pediatrics
2	0.21	Melatonin in children with autism spectrum disorders: endogenous and pharmacokinetic profiles in relation to sleep	3.2	Journal of Autism and Developmental Disorders
3	0.19	Sleep differences among children with autism spectrum disorders and typically developing peers: a meta-analysis	1.8	Journal of Developmental and Behavioral Pediatrics
4	0.17	Practitioner Review: Treatment of chronic insomnia in children and adolescents with neurodevelopmental disabilities	6.8	Journal of Child Psychology and Psychiatry
5	0.17	Clinical Practice Guideline for the Treatment of Intrinsic Circadian Rhythm Sleep-Wake Disorders: Advanced Sleep-Wake Phase Disorder (ASWPD), Delayed Sleep-Wake Phase Disorder (DSWPD), Non-24-Hour Sleep-Wake Rhythm Disorder (N24SWD), and Irregular Sleep-Wake Rhythm Disorder (ISWRD). An Update for 2015: An American Academy of Sleep Medicine Clinical Practice Guideline	3.5	Journal of Clinical Sleep Medicine

### 3.9 Analysis of keywords with the strongest citation bursts

Twenty-four sudden terms were found by performing an abrupt analysis of the keywords in the chosen literature (**Figure 7B**): Keywords like “autism spectrum disorder” (intensity 3.36), “circadian rhythm phase” (intensity 2.59), and “suprachiasmatic nucleus” (intensity 2.4) emerged between 2015 and 2017, indicating a growing interest among researchers in the connection between circadian rhythm mechanisms and neurological disorders. From 2016 to 2019, the increasing depth of clinical application research was reflected in the persistently high heat of keywords like “neurodevelopmental disorders” (intensity 3.54) and “sustained-release melatonin” (intensity 3.12). A trend towards the integration of sleep medicine with oncology, psychiatry, and pain medicine is evident in the rise of new hotspots like “mental disorders” (intensity 3.48), “breast cancer” (intensity 3.53), and “chronic pain” (intensity 2.45) after 2020.

## 4 Discussion

### 4.1 Status of the study

A total of 4,854 authors contributed to the 1,084 publications from 428 countries, 2,465 institutions, and 488 journals that were examined in this study. According to the analysis, there are three primary stages to annual publications. A comparatively small number of publications were published during the first phase, which ran from 2015 to 2018. The majority of the research was centered on controlled trials investigating the potential benefits of melatonin for treating insomnia as well as the relationship between melatonin and insomnia. To evaluate the therapeutic efficacy of melatonin, randomized controlled trials were carried out on a range of populations, including adults, children, the elderly, and perimenopausal women. The number of publications stabilized and exhibited an overall upward trend during the second phase, which lasted from 2019 to 2022. By measuring melatonin secretion levels, the primary research found that acupuncture, traditional Chinese medicine, and cognitive behavioral therapy were effective in treating insomnia. The third phase, which lasted from 2022 to 2024, saw a high volume of publications. The year with the most publications was 2022. By 2022, melatonin’s effectiveness as a treatment for insomnia had been mainly established; however, more research was required to determine whether it applied to patients with particular illnesses. A comparable number of publications were published in 2023 and 2024, with more researchers concentrating on the connection between melatonin and insomnia in relation to neurological disorders.

Geographically, the United States accounted for 27.7% of all publications with 300 publications, and the country had the highest total number of citations with 8,153. With 167 publications—or 15.4% of all publications—China came in second, with a total citation rate of 2,091, demonstrating the US’s considerable influence in this area.

The Institutional Collaboration Network reports that two of the top 10 institutions are from the United States, two are from the United Kingdom, two are from Italy, and two are from Australia. In comparison, although China ranks high in the total number of publications in this research field, the number of publications from each institution is relatively low, indicating a lack of continuity among Chinese institutions in the research on the correlation between melatonin and insomnia. Sapienza University of Rome, Harvard Medical School, and Monash University are the top three universities. It is important to remember that hospitals and universities make up the majority of these institutions. It is worth noting that Sapienza University of Rome is located in Italy, which ranks fourth in terms of publication volume, indicating that the University of Rome has extensibility in this field of research. In the citation analysis, the University of California, San Francisco ranks first with 1,079 citations, but only seven publications, indicating that this institution has high-quality publications.

Following an analysis of the authors, Bruni O is in the lead with 17 published pieces, followed by Palagini L in second place with 15 articles, and Ferri R and Zisapel N in a tie for third place with 11 articles apiece. The majority of Bruni O’s published works discuss children and examine how well melatonin works to cure sleeplessness in young patients. In his essays, Bruni O analyzes childhood insomnia from several angles, including its main symptoms, diagnosis, and treatment options. 97.4% of pediatricians prescribe melatonin to children with chronic insomnia, with 87.3% of patients being between the ages of one and two, according to an online poll on the topic. Of the patients, 42.5% were between the ages of 10 and 18, and 62.1% were between the ages of 2 and 5 (13). The main focus of author Palagini L’s work was creating professional practice recommendations for hypnotic drugs. She also employed systematic reviews to examine the effectiveness of melatonin in treating adult insomnia. The classification of insomnia and its physiological and pathological phases were the main topics of author Ferri R’s research. She summarized the effectiveness of medicine and cognitive behavioral therapy as treatments for insomnia. It was discovered that Ferri R and Palagini Lworked closely together, and their research topics were very relevant to one another.

An analysis of the results of co-cited authors revealed that Bruni O, Malow B.A, Gringras P, and Cortese S were the next most frequently mentioned authors. The main topic of author Zisapel N’s publications is melatonin’s function in regulating circadian rhythms and human sleep. The study cohort comprised children with neurodevelopmental problems, people with Alzheimer’s disease (AD), and those with jet lag from shift work (13). While children are the primary focus of Malow Beth A’s melatonin study, she also points out that sleep problems are linked to a number of neurological illnesses and are frequently brought on by the overlapping interplay of genetic, neurobiological, and environmental variables. Insomnia or hypersomnia are common symptoms of these diseases (14). Assessments of children with autism spectrum disorders’ behavioral skills and quality of life, as well as the effectiveness of melatonin in treating sleep issues, are the main areas of research for author Gringras P. Children with attention-deficit/hyperactivity disorder (ADHD) and teenagers with neurodevelopmental issues can benefit from melatonin treatment, according to Cortese S’s major research on the topic.

Sleep Medicine is the most prolific journal, according to journal analysis, with 29 articles and 680 citations overall. This suggests that the publication has made significant contributions to the study of melatonin and insomnia. With 2,310 citations, SLEEP is the most referenced journal. With 92.47 average citations, the Journal of Sleep Research has the most citations of any journal. Frontiers in Psychiatry, Journal of Clinical Sleep Medicine, Journal of Sleep Research, Sleep Medicine Reviews, International Journal of Circadian Rhythms, Nature and Science of Sleep, Sleep, International Journal of Environmental Research and Public Health, and Journal of Affective Disorders are the top ten journals in terms of publication frequency. Despite not having the highest publishing volume, Sleep Medicine Reviews (IF = 11.2), which is categorized as Q1 in the JCR, has the highest average number of citations among them, suggesting that higher article quality is associated with higher citation rates. This provides melatonin therapy for insomnia with a strong theoretical underpinning and data study background. With impact factors greater than 5, the top three journals in terms of publishing frequency are Journal of Clinical Sleep Medicine (IF = 3.5), Frontiers in Psychiatry (IF = 3.2), and SLEEP MEDICINE (IF = 3.8). This implies that the caliber of publications in this field of study needs to be raised, and more focused research ought to be done in order to publish in reputable journals and increase their impact.

According to the frequency of citations, the top five references were Gringas P. (2017), Sateia MJ. (2017), Maras A. (2018), Riemann D. (2017), Zisapel N. (2018), and Bruni O. (2015). We discovered that the top five papers among these comprise evaluations of the current state of melatonin use, clinical practice guidelines, and clinical trial research. More impartial proof of melatonin’s effectiveness in treating insomnia can be found in clinical trial studies. In order to treat insomnia in children and adolescents with autism spectrum disorder (ASD), a unique formulation of prolonged-release melatonin minitablets (PedPRM) that is appropriate for pediatric use was tested with a placebo in (Gringas P, 2017). PedPRM was reported to be safe and effective for treating insomnia in children and adolescents with ASD after 13 weeks of double-blind treatment, regardless of whether they also had neurodevelopmental disorders (NDDs) or attention deficit hyperactivity disorder (ADHD) (15). Melatonin drugs have been included in clinical practice recommendations for the treatment of adult chronic insomnia, according to (Sateia MJ, 2017), and cognitive behavioral therapy has been established as the standard treatment strategy in addition to medication (16). A sleep history, the use of sleep questionnaires and sleep diaries, and other moderate- to high-quality evidence should all be considered in the diagnosis of insomnia, according to Maras (2018). Adults with chronic insomnia are advised to begin treatment with cognitive behavioral therapy (17). Cognitive behavioral therapy is mentioned in both guidelines, suggesting that it has emerged as the mainstay of treatment for insomnia. Melatonin, a circadian rhythm cue, has been shown to impact circadian rhythms, restore sleep duration in older adults, and encourage sleep anticipation in the brain’s default mode network (DMN) (Zisapel N, 2018). In patients with Alzheimer’s disease (AD), exogenous melatonin can enhance cognitive decline and cardiovascular health (16). Melatonin’s usage in treating pediatric mental illnesses is the main topic of discussion in Bruni O. (2015). It has been observed that children are now the main demographic impacted by insomnia (18). Notably, Gringas P (2017), and Riemann D (2017), both of whom had impact factors greater than 5, showed that exogenous melatonin has a substantial therapeutic effect on insomnia, offering excellent theoretical justification for its use in treating insomnia.

Chang YS, 2016 (0.29), Goldman SE, 2014 (0.21), Elrod MG, 2015 (0.19), Bruni O, 2018 (0.17), and Auger RR, 2015 (0.17), are the top five centrality rankings of the mentioned references. The article at the center of the cited literature is indicated with the highest centrality. Based on a randomized clinical trial, the cited reference Chang YS (2016) showed that melatonin administration is a safe and efficient way to improve children with Atopic Dermatitis (AD)’s sleep latency and disease severity (19). With an Impact Factor (IF) of 24.7 and a ranking in the top quartile (Q1) of the Journal Citation Reports (JCR), this article’s publication in JAMA Pediatrics demonstrated its authority. Melatonin’s function in children with ASD was examined by Goldman SE, 2014 (0.21) and Elrod MG, 2015 (0.19), both of which confirmed that ASD is a mental illness linked to symptoms of insomnia (20, 21). Melatonin is the safest alternative for children with NDDs, according to growing data (22). The American Academy of Sleep Medicine’s Clinical Practice Guidelines support the use of timed melatonin in treating children and adolescents with delayed sleep-wake phase disorder (DSWPD), but more research is required to determine its suitability for use in adults and the elderly (Auer RR, 2015, 0.17). Notably, three diseases—ASD, NDDs, and DSWPD—are included in the top five most cited references. All of these illnesses are linked to sleeplessness in one way or another, and insomnia is very common.

### 4.2 Analysis of research hotspots

#### 4.2.1 Diseases and populations for melatonin application

Since sleeplessness is closely linked to Parkinson’s disease (PD), Alzheimer’s disease (AD), and autism spectrum disorder (ASD), melatonin research has focused on these conditions. Neurodegenerative illnesses like AD and PD mainly impact the elderly. Melatonin therapy can be seen as a causative treatment method in pediatric populations at high risk for sleeplessness, as children are the primary population affected by ASD (23). According to research, children’s melatonin intake has increased by 530% to 600%, and the number of young people using prescription melatonin has increased dramatically in countries including Denmark, Norway, Sweden, the United States, and Australia (24). Melatonin has shown outstanding safety in this particular kid population in clinical trials. Approximately 87% of doctors have prescribed melatonin for children, according to clinical studies. Common dosages are 3 or 6 mg, and no serious side effects have been reported (25). Among children with ASD, melatonin is one of the main treatments for sleeplessness, which affects 50–80% of them (26–28). For instance, administering 3 mg of melatonin orally before bed can reduce screaming bouts considerably, increase the quality of sleep (29), and relieve non-motor symptoms while remaining safe (30).

The effects of melatonin go beyond controlling circadian cycles. It has been shown to scavenge free radicals, enhance mitochondrial function, and alter immune system responses, which eventually slows the course of neurodegenerative illnesses (31) by lowering sleep latency and increasing sleep length (15, 32). Despite the fact that the melatonin cycle is not thought to be an intrinsic part of the fundamental circadian clock mechanism, using circadian rhythm output channels for medication therapy would be a more beneficial strategy (33, 34). Melatonin has been shown in clinical investigations to improve sleep in AD patients by lowering tau hyperphosphorylation and controlling amyloid-β (Aβ)-induced neuroinflammation (35). Treatment of melatonin for sleep disturbances in Parkinson’s disease: According to a meta-analysis and comprehensive review, melatonin may be a useful treatment for PD patients’ insomnia (36), sleep quality, and daytime drowsiness (37).

#### 4.2.2 The mechanism by which melatonin is applied to insomnia

Three main mechanisms underlie melatonin’s usefulness in treating insomnia: pineal receptor activity, gut-brain axis connection, and circadian rhythm control. The pineal gland in the brain secretes the hormone melatonin, which is essential for preserving circadian rhythms and the sleep-wake cycle (38). Through the melatonin receptors MT1 and MT2, it affects the sleep-wake cycle and has a role in controlling the organization and quality of sleep (39). Higher melatonin dosages are needed if melatonin receptor sensitivity declines. Insomnia is intimately linked to the disruption of core circadian clock genes (e.g., CLOCK, BMAL1, and PER/CRY), which use the SCN to govern peripheral tissue rhythms (40–43). Changes in the gut microbiota’s composition and rhythm are linked to circadian rhythm problems, and research has shown that the two are interacting (44). GABA, tryptophan, and short-chain fatty acids are just a few of the metabolites that the gut microbiota can use to affect the central nervous system. Sleep is impacted by ecological dysbiosis because it inhibits melatonin precursor absorption, 5-HT synthesis, pineal gland function, and melatonin release. In order to cure insomnia, melatonin should be applied using a multi-target intervention strategy that takes into account gut-brain axis regulation, receptor function restoration, and circadian rhythm synchronization.

### 4.3 Research trend analysis

Current research trends are somewhat reflected in the examination of the quantity of citations for keywords in this field. Future study in this area will likely focus on the integration of insomnia with pain management, psychology, and oncology. Surveys show that between 29 and 75 percent of cancer patients suffer from sleeplessness, which lasts the duration of the illness and treatment (45). The bidirectional relationship between insomnia and cancer can now be explained by a number of molecular mechanisms, such as hypothalamic-pituitary-adrenal axis dysfunction and cytokine dysregulation (46). As a result, addressing insomnia benefits both long-term cancer survivors and people receiving ongoing cancer treatment. This reciprocal association raises the possibility that prompt sleep intervention could be a novel cancer treatment target (47). According to studies, melatonin supplements can help breast cancer patients with their sleep issues while also increasing the sensitivity of chemotherapy medications and lowering their adverse effects (48). Melatonin can help with sleeplessness in neurological conditions such as Alzheimer’s disease (AD), Parkinson’s disease (PD), epilepsy, stroke, and restless legs syndrome (RLS). It is also involved in neuroprotection, mood modulation, antioxidant activity, and other brain activities (49–52). Up to 48% of people with chronic sleep difficulties may have sleep disorders (53). While poor sleep quality might worsen sleep-related pain and pain spread (54), research shows that long-term treatment of sleep problems can alleviate physical pain and may avoid chronic pain disorders in insomnia patients (55). Melatonin enhances analgesic effects by blocking nociceptive signal transmission through MT1/MT2 receptors in the spinal dorsal horn. Melatonin and its receptor agonists have demonstrated good efficacy in treating insomnia in fibromyalgia, according to clinical investigations, underscoring their potential to reduce pain, promote better sleep, and improve quality of life (56). To fully utilize melatonin’s therapeutic potential in neurological illnesses, pain-related problems, and oncological diseases, future studies should concentrate on how it works in concert with other forms of treatment. Research tactics should include encouraging high-quality systematic reviews that incorporate evidence and guide practice and supporting international multi-center collaborative projects. The pathophysiological pathways that sleeplessness and other comorbidities share should be further studied. High-quality data should be concentrated on certain populations, such as older people with neurodegenerative diseases and children with neurodevelopmental abnormalities, when converting findings into therapeutic practice. Instead of replacing CBT-I, melatonin should be marketed as an additional or supplemental treatment. Stress that melatonin should be used appropriately in clinical settings as a “circadian rhythm regulator” as opposed to a “sleep aid.” Regulatory bodies could think about turning melatonin into a medicine for management and enforcing stringent labeling laws to guarantee precise dosage and clear information. Government departments should also set up a nationwide monitoring system while formulating policies.

## 5 Advantages and limitations

Benefits: In order to find pertinent papers published within the last ten years, this study searched the WOS Core Collection and PubMed databases with an emphasis on the relationship between melatonin and insomnia. It carried out a thorough examination of the nations, organizations, journals, writers, and keywords connected to these pieces. Based on a timetable, the study examined the current research hotspots and upcoming trends in this subject using keyword clustering and the top 25 most popular phrases. It offers a thorough examination of the connection between melatonin and insomnia from a variety of angles, as well as theoretical justification for melatonin use and insomnia treatment.

Drawbacks:

(1) Only articles from the last ten years were found; hence, the time period of the literature search in this paper is not entirely covered.
(2) Chinese-language databases were not included in this work; only English-language databases were.
(3) Only the English language is utilized; no other languages are employed.
(4) Gray literature was not included because conference proceedings, abstracts, editorial materials, letters, collections, anthologies, news articles, book chapters, and retracted publications were not included.
(5) Only co-authorship was used to determine institutional collaborations, which prevented the analysis of individual relationships.
(6) Positive and critical citations cannot be distinguished by co-citation relationships.
(7) Despite the fact that this study’s bibliometric analysis showed the academic activity and influence of various nations, organizations, and researchers in the field of melatonin and insomnia research, these indicators primarily reflect research interest and academic influence and cannot be used as direct proof of the clinical effectiveness of melatonin.

## 6 Summary

Using bibliometric techniques, a thorough review of studies on the use of melatonin to treat insomnia from 2015 to 2024 allows for the following main findings to be made:Research in this area has been steadily increasing in recent years, with the majority of these efforts being focused on industrialized nations in the US and Europe. Among them, the United States is the leader in both publications and total citations, whereas China might yet do better in terms of influence despite having a large number of publications. Melatonin applications are targeted at particular demographics (e.g., elders and children) and comorbid conditions (e.g., neurological disorders), according to research hotspots. Melatonin in particular shows promising safety and potential efficacy in treating insomnia linked to Parkinson’s disease, Alzheimer’s disease, and autism spectrum disorders. Its main modes of action are multi-pathway synergistic effects, including modulation of receptor function, gut-brain axis interaction, and circadian rhythm regulation. Future studies will concentrate on comorbid processes and multimodal treatment approaches, further integrating other disciplines, including pain medicine, mental illnesses, and oncology. Standardizing clinical applications, enhancing international collaboration, and bolstering high-quality clinical research are also necessary to properly utilize melatonin’s all-encompassing benefits in treating insomnia.

## Data Availability

The original contributions presented in the study are included in the article/**Supplementary Material**. Further inquiries can be directed to the corresponding authors.
