# Supplementary material for: Status of research on the application of melatonin in insomnia based on bibliometric visualization analysis and development trends
Source: Front Psychiatry. 2025 Sep 29;16:1640198. doi: 10.3389/fpsyt.2025.1640198 (PMC12515902; doi:10.3389/fpsyt.2025.1640198)
Supplement: Supplementary file 1 [file Table1.docx]

Supplementary Material

# Supplementary Tables 1

Detailed Search Strategy Table

| Database | Search term |
| --- | --- |
| Web of Science core collection | #1 TS=(“Disorders of Initiating and Maintaining Sleep” or “Disorders of Initiating and Maintaining Sleep” or “Sleeplessness” or “Insomnia Disorder” or “Insomnia Disordersor” or “Insomnias” or “Chronic Insomnia” or “Insomnia, Chronic” or “Early Awakening” or “Awakening, Early” or “Nonorganic Insomnia” or “Insomnia, Nonorganic” or “Primary Insomnia” or “Insomnia, Primary” or “Psychophysiological Insomnia” or “Insomnia, Psychophysiological” or “Rebound Insomnia” or “Insomnia, Rebound” or  “Secondary Insomnia” or “Insomnia, Secondary” or “Sleep Initiation Dysfunction” or “Dysfunction, Sleep Initiation” or “Dysfunctions, Sleep Initiation” or “Sleep Initiation Dysfunctions” or “Transient Insomnia” or “Insomnia, Transient”) |
|  | #2 TS="melatonin" |
|  | #3 #1 AND #2 |
| PubMed | #1 “Insomnia” [Mesh] |
|  | #2 “Disorders of Initiating and Maintaining Sleep” [Title/Abstract] OR “Disorders of Initiating and Maintaining Sleep” [Title/Abstract] OR “Sleeplessness” [Title/Abstract] OR “Insomnia Disorder” [Title/Abstract] OR “Insomnia Disordersor” [Title/Abstract] OR “Insomnias” [Title/Abstract] OR“Chronic Insomnia” [Title/Abstract] OR “Insomnia, Chronic” [Title/Abstract] OR “Early Awakening” or “Awakening, Early” [Title/Abstract] OR “Nonorganic Insomnia” [Title/Abstract] OR “Insomnia, Nonorganic” [Title/Abstract] OR “Primary Insomnia” [Title/Abstract] OR “Insomnia, Primary” [Title/Abstract] OR “Psychophysiological Insomnia” [Title/Abstract] OR “Insomnia, Psychophysiological” [Title/Abstract] OR “Rebound Insomnia” [Title/Abstract] OR “Insomnia, Rebound” [Title/Abstract] OR“Secondary Insomnia”[Title/Abstract] OR “Insomnia, Secondary”[Title/Abstract] OR “Sleep Initiation Dysfunction”[Title/Abstract] OR “Dysfunction, Sleep Initiation”[Title/Abstract] OR “Dysfunctions, Sleep Initiation” [Title/Abstract] OR “Sleep Initiation Dysfunctions”[Title/Abstract] OR “Transient Insomnia”[Title/Abstract] OR “Insomnia, Transient”[Title/Abstract] |
|  | #3 #1 OR #2 |
|  | #4 "melatonin"[Title/Abstract] |
|  | #5 #3 AND #4 |
